# Investigation of Optimal Light Source Wavelength for Cuffless Blood Pressure Estimation Using a Single Photoplethysmography Sensor

**DOI:** 10.3390/s23073689

**Published:** 2023-04-02

**Authors:** Sogo Toda, Kenta Matsumura

**Affiliations:** 1 Ishikawa College, National Institute of Technology, Tsubata 929-0392, Japan; 2 Faculty of Medicine, University of Toyama, Toyama 930-0194, Japan

**Keywords:** blood pressure, heart rate, modified normalized pulse volume, optical wavelength, photoplethysmography

## Abstract

Routine blood pressure measurement is important for the early detection of various diseases. Recently, cuffless blood pressure estimation methods that do not require cuff pressurization have attracted attention. In this study, we investigated the effect of the light source wavelength on the accuracy of blood pressure estimation using only two physiological indices that can be calculated with photoplethysmography alone, namely, heart rate and modified normalized pulse volume. Using a newly developed photoplethysmography sensor that can simultaneously measure photoplethysmograms at four wavelengths, we evaluated its estimation accuracy for systolic blood pressure, diastolic blood pressure, and mean arterial pressure against a standard cuff sphygmomanometer. Mental stress tasks were used to alter the blood pressure of 14 participants, and multiple linear regression analysis showed the best light sources to be near-infrared for systolic blood pressure and blue for both diastolic blood pressure and mean arterial pressure. The importance of the light source wavelength for the photoplethysmogram in cuffless blood pressure estimation was clarified.

## 1. Introduction

High arterial blood pressure (BP) is known worldwide as a risk factor for various diseases, including cardiovascular disease [1,2,3]. Because BP can change significantly in a short period of time, it is important to measure it frequently for routine health monitoring [4,5]. Therefore, routine BP measurement methods must be noninvasive and simple. Currently, the most widely used BP measurement method is the cuff-based BP measurement method, which is based on either the oscillometric method [6] or the Riva-Rocci–Korotkov method [7]. Although cuff-based BP measurement is noninvasive and highly accurate, the cuff must be attached to the upper arm, wrist, or fingers and then pressurized; this causes discomfort during measurement and means that the method is unsuitable at certain times, including during sleep.

To address this problem, “cuffless BP”—a method of BP estimation that does not require cuff pressurization—has been explored [8,9,10,11,12,13,14,15,16,17,18,19,20,21,22,23,24,25]. The advantages of cuffless BP are that it eliminates the inconvenience and discomfort of pressurization and does not require a pressurization device, thereby allowing the measurement device to be simplified and downsized. These advantages make cuffless BP suitable for measurement at any time. Various methods have been proposed for cuffless BP estimation, including methods based on pulse transit time (PTT) [8,9,10,11,12,13,14,15,16,17,18]; methods using feature values of PTT, photoplethysmograms (PPGs), and electrocardiograms (ECGs); and methods using pressure sensors and PPG [19,20,21,22,23,24,25]. Typical PTT-based methods include those that perform PPG measurements at two different locations to obtain the PTT and those that use the time shift between the ECG and PPG peaks [9]. It is known that there is a positive correlation between PTT and BP [26]. To estimate BP from the PTT, there are methods that approximate the relationship between them using a linear model [10], a logarithmic model [11], or an inverse-square model [12]; a method using the Hilbert–Huang transform used in signal processing [13]; and a nonlinear second-order curve-fitting regression model [14]. All of these methods require calibration using a cuff sphygmomanometer [17,18]. Other cuffless BP estimation methods include those that estimate BP from ECG and PPG features [19,20] and those that estimate BP from PPG features alone with machine learning [21,22]. Moreover, the method of using a pressure sensor and a PPG requires the fingertip to be pressed into the pressure sensor, which makes the measurement burdensome [23].

Instead, we have studied a method for estimating BP from a single PPG based on findings from circulation physiology and psychophysiology [24,25]. This method uses the fact that BP is expressed as the product of cardiac output (CO) and total peripheral resistance (TPR) [27]. Furthermore, the CO and TPR required for BP estimation can be approximated using the heart rate (HR) and the modified normalized pulse volume (mNPV) obtained from the PPG, respectively [28,29,30,31,32]. Therefore, BP can be estimated from the product of HR and mNPV. Using this method, BP is estimated by calculating the mNPV and HR from the obtained PPG and so can be realized using a single PPG, and thus, BP can be estimated with a certain degree of accuracy using only the built-in flashlight and CMOS camera of a smartphone [25].

The proposed method performs BP estimation using a single PPG, but in previous studies, the light source used for PPGs has generally been restricted to near-infrared (NIR) light [25]. NIR and green light are the most common light sources used for PPGs, but the optical properties of biological tissues mean that the longer the wavelength, the deeper the penetration into the body and the different tissues that are passed through [33,34,35]. Therefore, it is possible that different light source wavelengths may cause different types of blood vessels to be observed. Although CO does not change depending on the vessels observed, TPR may change. Thus, the estimated BP may differ depending on the wavelength used. Therefore, it is necessary to investigate the wavelength characteristics of the PPG light source for the proposed method in order to study the fundamental characteristics of this method. BP estimation at multiple PPG wavelengths can provide information on the optimal wavelength, but BP can vary widely from second to second. Therefore, we measured PPGs at multiple wavelengths simultaneously and used them to estimate BP using the proposed method. Our aim was to investigate the optimal wavelength by comparing the estimated BP with that measured using a cuff sphygmomanometer. This is the first study to focus on the wavelength used for PPGs in cuffless BP estimation.

## 2. Materials and Methods

### 2.1. PPG Measurement

Photoplethysmography involves obtaining waveforms of changes in the volume of blood vessels by using the optical absorption characteristics of blood [36,37]. It is performed using a noninvasive and compact device because measurements are made using only a light source and a photodetector. There are 2 PPG measurement modes: the reflectance mode, in which the light source and photosensor are placed on the same side of the measurement site, and the transmittance mode, in which they are placed on opposite sides. The PPG measurement site is a fingertip, wrist, or earlobe.

**Figure 1:**
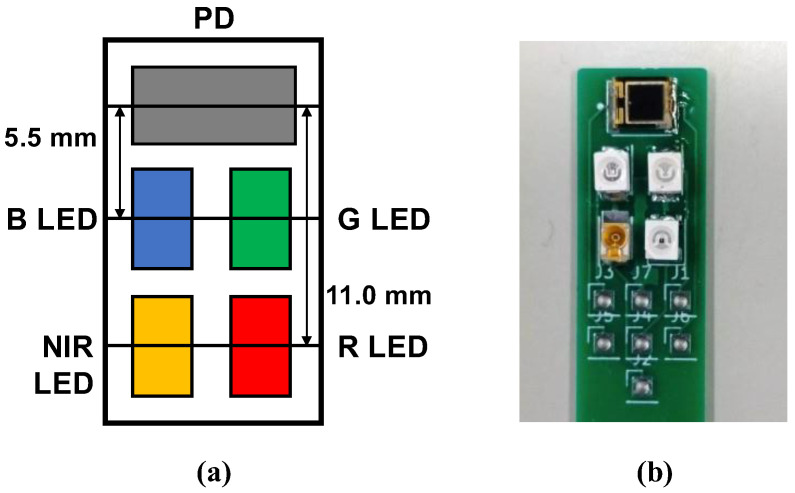
Developed multiwavelength photoplethysmogram (PPG) sensor: (**a**) layout of light-emitting diodes (LEDs) and photodiode (PD). B LED = blue LED (470 nm), G LED = green LED (528 nm), R LED = red LED (620 nm), and NIR LED = near-infrared LED (870 nm); (**b**) actual PPG sensor.

**Figure 2:**
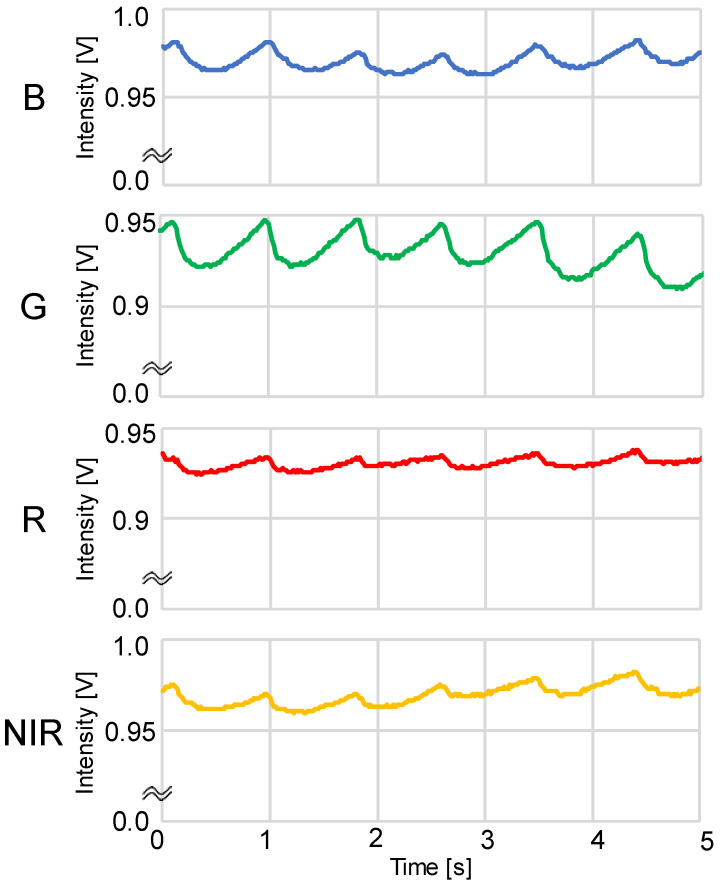
Example of simultaneous recordings of blue, green, red, and near-infrared light photoplethysmograms (PPGs) measured using a DC amp: B = blue LED PPG, G = green LED PPG, R = red LED PPG, and NIR = near-infrared LED PPG.

In this study, we constructed a multiwavelength PPG sensor, as shown in Figure 1, to measure multiwavelength PPGs simultaneously. For the light sources, we used 4 light-emitting diodes (LEDs), namely, an NIR LED (870 nm; SIM-040ST), a red LED (620 nm; SML-Z14U4T), a green LED (528 nm; SMLZ14EGT), and a blue LED (470 nm; SMLZN4BGT) (all from ROHM Semiconductor, Kyoto, Japan), and as the photodetector, we used a photodiode (TEMD5510; Vishay Semiconductors, Malvern, PA, USA). Because blue and green cannot be measured in transmittance mode, our developed PPG sensor operates in reflectance mode. As shown in Figure 2, to measure multiwavelength PPGs simultaneously, the 4 LEDs were chopper-emitted, and the light intensity of each was adjusted so that it was the same after propagation through the fingertip.

**Figure 3:**
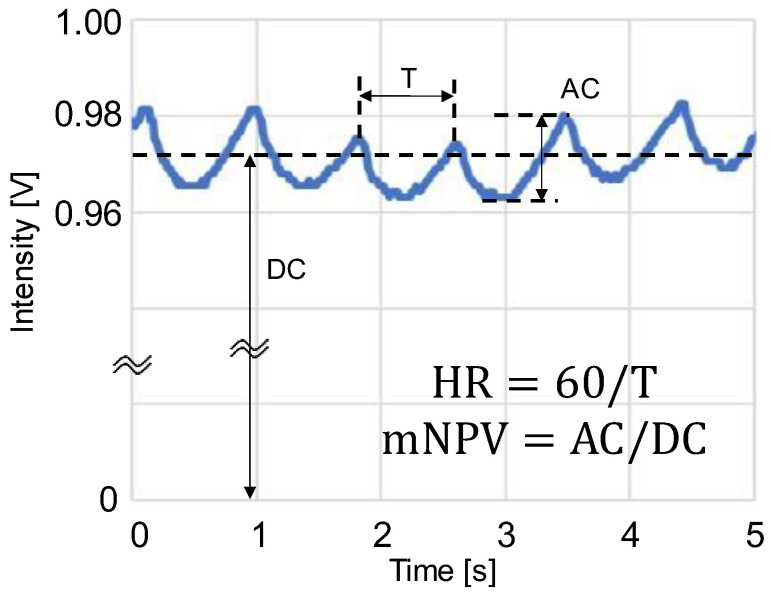
Schematic of photoplethysmogram (PPG) waveform feature values: AC is the amplitude of PPG, DC is the mean value of PPG, T is the peak interval time, HR = heart rate, and mNPV = modified normalized pulse volume [31].

The PPGs were obtained by sampling at a frequency of 60 Hz, performing analog-to-digital conversion using a microcontroller (mbed LPC1768; ARM, Cambridge, UK), and then importing the data into a computer. The PPG waveforms were processed as shown in Figure 3 using the LabVIEW 2020 software (version 20.0f1 (32-bit)), and the AC component, DC component, and peak interval (T) of the waveforms were averaged to obtain the HR and mNPV. The HR was obtained from the peak interval T of the PPG as HR = 60/T, and the mNPV was calculated from the AC/DC ratio of the PPG. During the waveform analysis, positions where maxima continued or where T was extremely short or long were removed as outliers.

### 2.2. Cuffless BP Estimation Method

BP is calculated using CO and TPR as BP = CO × TPR [27]. Taking the natural logarithm (ln) of both sides of this equation gives ln BP = ln CO + ln TPR, and so BP can be regarded as a simple linear polynomial. Furthermore, CO and HR are correlated because they are both affected by β-adrenergic sympathetic nerve activity [28,29]. Similarly, TPR and mNPV are correlated because they are both affected by α-adrenergic sympathetic nerve activity [30,31,32]. Therefore, the equation

(1) lnBP=lnHR+lnmNPV

holds, which shows that BP can be estimated using only the HR and mNPV obtained from a single PPG measurement [25]. The same idea applies to systolic BP (SBP), diastolic BP (DBP), and mean arterial pressure (MAP).

### 2.3. Experimental Protocol

In this study, we investigated how the wavelength of the light source affects the proposed method by measuring BP simultaneously with both a cuff sphygmomanometer and the 4-wavelength PPGs obtained using the multiwavelength PPG sensor shown in Figure 1.

The experiment was conducted in a conference room where the temperature was kept at 20–24 °C. Each participant sat at rest in a chair with both hands on a desk in front of them. A PPG sensor was attached to the index finger of each participant’s left hand, and a cuff sphygmomanometer (HCR-7601T; OMRON Healthcare, Kyoto, Japan) was attached to their upper right arm. The PPG sensor was attached to the tip of the participant’s index finger using tape to ensure that all 4 LEDs and the PD were in contact with the finger without loosening and that the participant did not feel pressure. The finger with the PPG sensor and the upper arm with the cuff were supported at heart level. The participant was instructed to remain as still as possible during the measurement to reduce the effects of motion artifacts.

**Figure 4:**
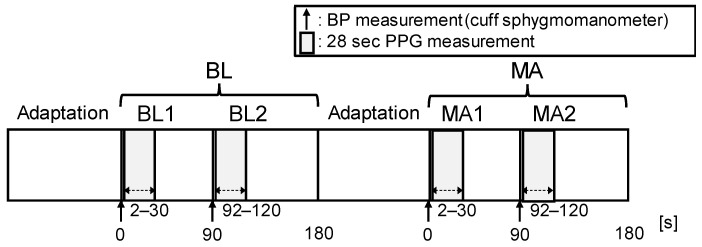
Experimental protocol. The experiment consisted of 2 periods: baseline (BL) and mental arithmetic (MA). Each period lasted 180 s. A total of 2 BP measurements and a PPG measurement (28 s) were taken for each period. The BL period measured the resting condition, and the MA period measured the stress condition induced via mental arithmetic.

As shown in Figure 4, the experiment began with a 3 min baseline (BL) measurement after a sufficient adaptation period. During BL, PPGs were measured constantly, and the cuff sphygmomanometer was used twice, once at the start of measurement and then 90 s thereafter. Next, the participant performed a 3 min mental arithmetic task (MA) in which they were instructed to continuously subtract a number (e.g., keep subtracting 13 from 3000) and to do it as quickly and accurately as possible. During the MA period, PPGs and BP were measured at the same times as they were during the BL period. To calculate the HR and mNPV for the BP estimation with the PPG, we used the PPG waveform from the start of the cuff-sphygmomanometer measurement to 30 s thereafter. However, because the PPG included motion artifacts due to cuff pressure, the waveform during the first 2 s of measurement was removed.

### 2.4. Participants

In total, 14 volunteers (6 Japanese women and 8 Japanese men, aged 19–21 years (19.4 ± 0.6 (mean ± standard deviation)) were recruited from within Ishikawa College and participated in this study. To do so, volunteers had to be at least 18 years old and free of cardiovascular disease at the time of the experiment. The BPs of the participants measured with a cuff sphygmomanometer are summarized in Table 1. According to the criteria of the American Heart Association [38], there were 2 participants with hypertension stage 1 (130/90 mmHg) and 2 participants with hypotension (90/60 mmHg). For participating, each participant received approximately USD 30. After explaining the details of this study to the participants, written informed consent was obtained. This study was approved by the ethics committee of the National Institute of Technology of Ishikawa College (IRB number: N.A.) on 13 November 2019 and was conducted in accordance with the principles expressed in the Declaration of Helsinki.

The sample size of this study was relatively small (*N* = 14) because previous studies on BP estimation based on pulse wave velocity and PPGs have shown that a small number of participants is acceptable unless there is a specific reason indicating otherwise, such as the use of machine learning. For example, Payne et al. [15] used *N* = 12, Patzak et al. [16] used *N* = 12, and the previous study on the proposed method used *N* = 13 [25].

### 2.5. Data Analysis

**Figure 5:**
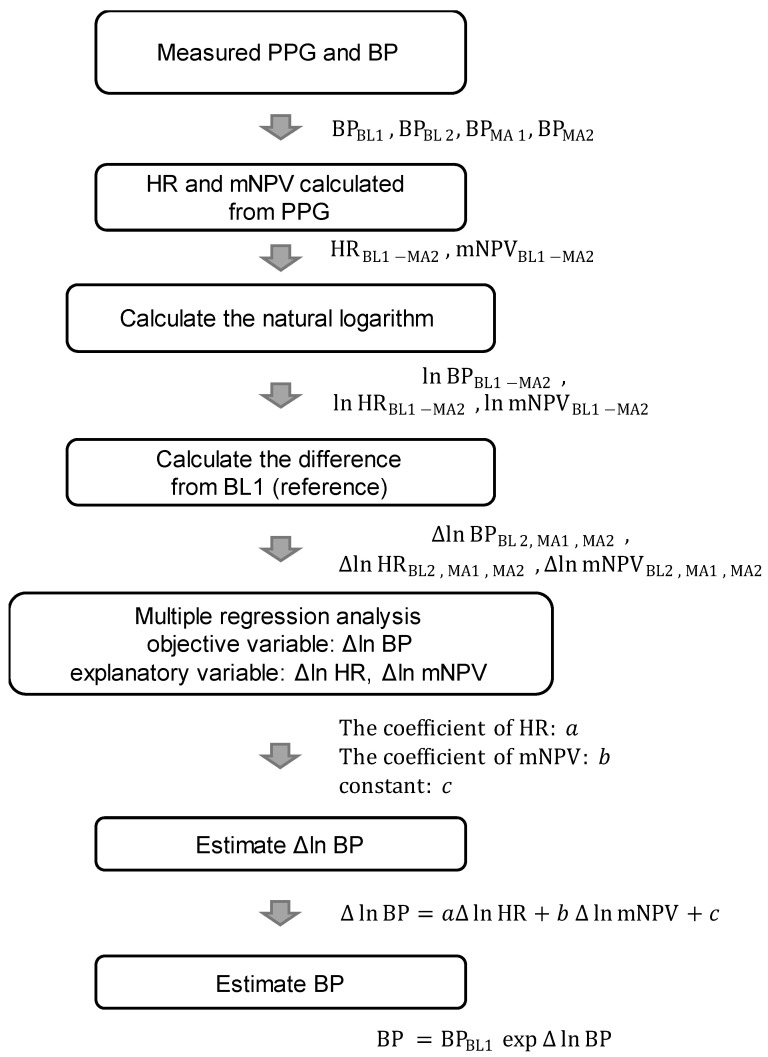
Flowchart of BP estimation. BP = blood pressure (SBP/DBP/MAP), HR = heart rate, and mNPV = modified normalized pulse volume.

Figure 5 shows a flowchart of BP estimation with this method. As shown in Figure 4, 4 BP measurements were taken per participant, 2 in BL (BL1 and BL2) and 2 in MA (MA1 and MA2). The HR and mNPV for each beat were calculated from the PPG waveform at 2–30 s from the start of the BP measurement and averaged. Then, the natural logarithms of HR (ln HR_BL1_, ln HR_BL2_, ln HR_MA1_, and ln HR_MA2_), mNPV (ln mNPV_BL1_, ln mNPV_BL2_, ln mNPV_MA1_, and ln mNPV_MA2_), SBP (ln SBP_BL1_, ln SBP_BL2_, ln SBP_MA1_, and ln SBP_MA2_), DBP (ln DBP_BL1_, ln DBP_BL2_, ln DBP_MA1_, and ln DBP_MA2_), and MAP (ln MAP_BL1_, ln MAP_BL2_, ln MAP_MA1_, and ln MAP_MA2_) for each period were calculated to normalize the distribution.

Furthermore, to suppress individual differences, the difference values (Δln HR, Δln mNPV, Δln SBP, Δln DBP, and Δln MAP) for each value of BL2, MA1, and MA2 based on the value of BL1 were calculated, respectively, and used in the data analysis (e.g., Δln SBP_MA1_ = ln SBP_MA1_ − ln SBP_BL1_ = ln (SBP_MA1_/SBP_BL1_)). Based on Equation (1), Δln SBP, Δln DBP, and Δln MAP are expressed as follows [25]:

(2) $$\mathrm{Estimated}\Delta \ln\mathrm{SBP}=a_{SBP}\Delta \ln\mathrm{HR}+b_{SBP}\Delta \ln\mathrm{mNPV}+c_{SBP},$$

(3) $$\mathrm{Estimated}\Delta \ln\mathrm{DBP}=a_{DBP}\Delta \ln\mathrm{HR}+b_{DBP}\Delta \ln\mathrm{mNPV}+c_{DBP},$$

(4) $$\mathrm{Estimated}\Delta \ln\mathrm{MAP}=a_{MAP}\Delta \ln\mathrm{HR}+b_{MAP}\Delta \ln\mathrm{mNPV}+c_{MAP}.$$

The values of the coefficients *a*, *b*, and *c* in Equations (2)–(4) were obtained using multiple linear regression analysis with Δln SBP, Δln DBP, and Δln MAP as the objective variables and Δln HR and Δln mNPV as the explanatory variables. Via the natural exponential transformation (exp) of Equations (2)–(4), the ratio of BP to the reference value BL1 was calculated (e.g., exp(Δln SBP_MA1_) = SBP_MA1_/SBP_BL1_). With this method, if the cuff sphygmomanometer value at BL1 is known, then the estimated BP for BL2, MA1, and MA2 can be obtained as the difference from the BP values obtained using Equations (2)–(4).

Therefore, by multiplying the known SBP_BL1_, DBP_BL1_, and MAP_BL1_ by the exponentially transformed estimated Δln SBP, Δln DBP, and Δln MAP, as in Equations (5)–(7), we obtain the estimated SBP, DBP, and MAP [25]:

(5) $$\mathrm{Estimated}\;\mathrm{SBP}={\mathrm{SBP}}_{\mathrm{BL}1}\exp\left(\mathrm{Estimated}\Delta \ln\mathrm{SBP}\right),$$

(6) $$\mathrm{Estimated}\;\mathrm{DBP}={\mathrm{DBP}}_{\mathrm{BL}1}\exp\left(\mathrm{Estimated}\Delta \ln\mathrm{DBP}\right),$$

(7) $$\mathrm{Estimated}\;\mathrm{MAP}={\mathrm{MAP}}_{\mathrm{BL}1}\exp\left(\mathrm{Estimated}\Delta \ln\mathrm{MAP}\right).$$

The estimation accuracy was evaluated by comparing the estimated values of SBP, DBP, and MAP with those measured with a cuff sphygmomanometer.

## 3. Results

Table 2 gives the mean values and standard deviations of the HR and mNPV obtained from the four measured PPG waveforms for each condition. These changes are statistically significant, which is reasonable because stress increases cardiac output and constricts peripheral blood vessels.

The results of the multiple linear regression analysis using the Δln MAP, Δln SBP, and Δln DBP measured with a cuff sphygmomanometer as the objective variables and the Δln HR and Δln mNPV calculated from the PPG at each wavelength as the explanatory variables are given in Table 3.

As shown in Table 3, when comparing each wavelength in terms of the coefficient of determination (*R*^2^), NIR shows the highest accuracy for Δln SBP, while blue shows the highest accuracy for both Δln DBP and Δln MAP. For Δln SBP, although NIR has the best accuracy, blue also has a high accuracy of *R*^2^ = 0.612. In addition, for Δln SBP and Δln MAP, the accuracy of *R*^2^ > 0.5 is excellent. Comparing each BP shows a trend of joint best accuracy for Δln SBP and Δln MAP, followed by Δln DBP. Shapiro–Wilk tests did not detect a strong violation of the normality of the distribution in the residuals (all *p*-values were >0.011).

**Figure 6:**
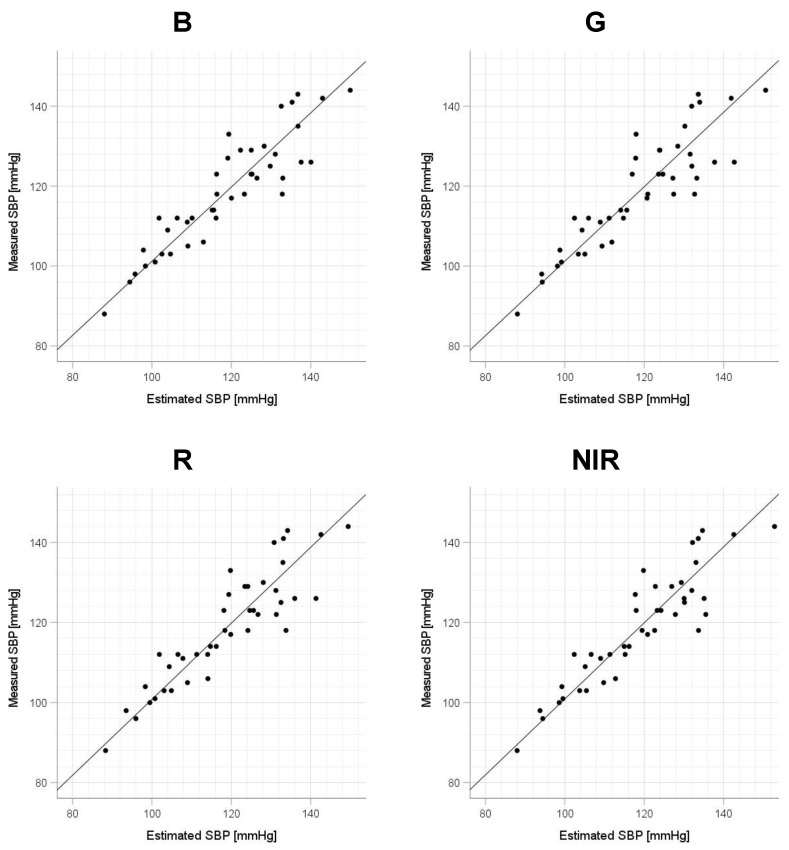
Accuracy of systolic blood pressure (SBP) estimation at each wavelength. Scatterplots of SBP estimated using proposed method and SBP measured with a cuff sphygmomanometer (*N* = 42). From left to right, the light source wavelengths are blue (B), green (G), red (R), and near-infrared (NIR). The solid line on each scatterplot is the regression line with geometric mean regression.

**Figure 7:**
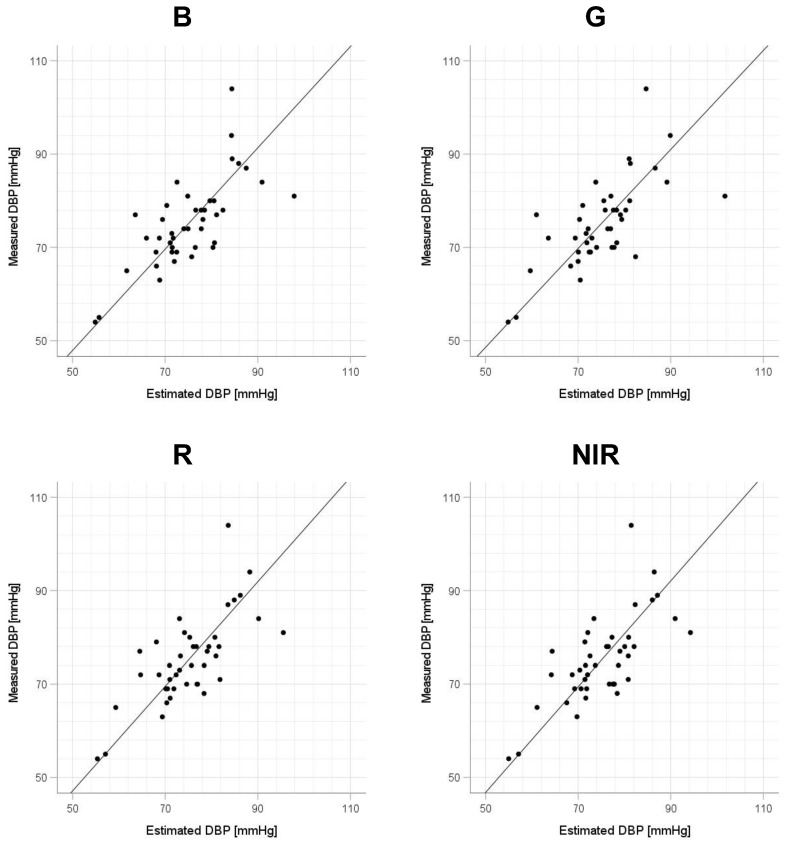
Accuracy of diastolic blood pressure (DBP) estimation at each wavelength. Scatterplots of DBP estimated using proposed method and DBP measured with a cuff sphygmomanometer (*N* = 42). From left to right, the light source wavelengths are blue (B), green (G), red (R), and near-infrared (NIR). The solid line on each scatterplot is the regression line with geometric mean regression.

**Figure 8:**
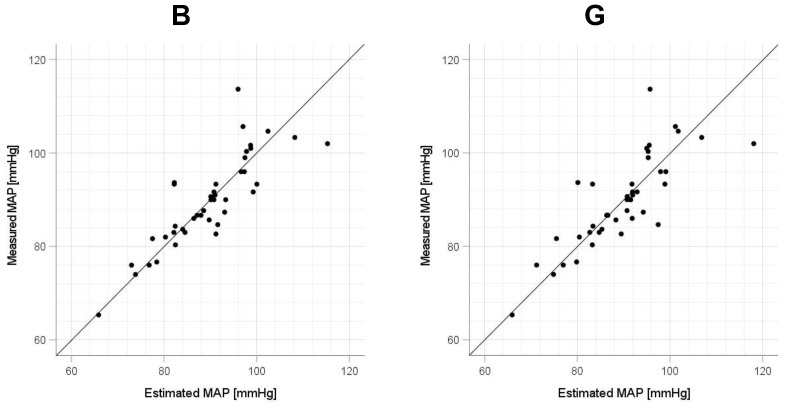

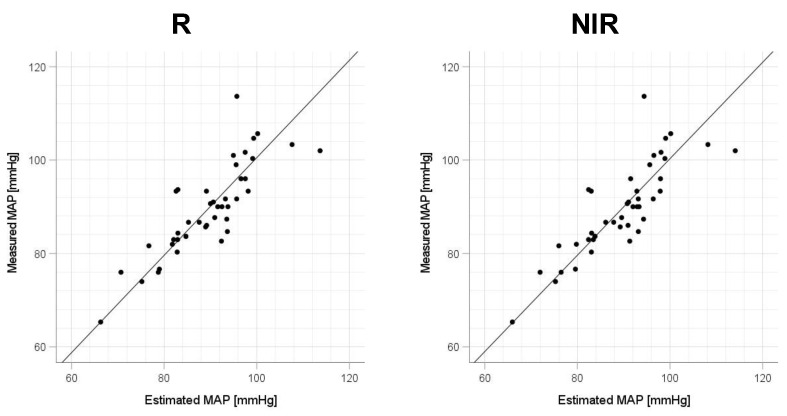
Accuracy of mean arterial pressure (MAP) estimation at each wavelength. Scatterplots of MAP estimated using proposed method and MAP measured with a cuff sphygmomanometer (*N* = 42). From left to right, the light source wavelengths are blue (B), green (G), red (R), and near-infrared (NIR). The solid line on each scatterplot is the regression line with geometric mean regression.

**Figure 9:**
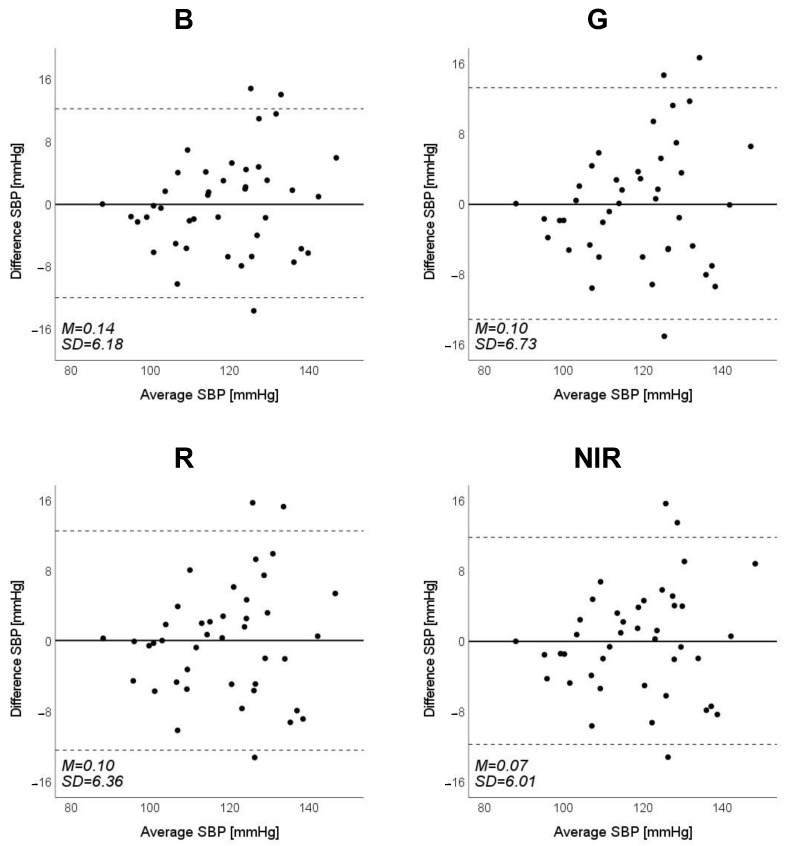
Bland–Altman plots of estimated systolic blood pressure (SBP) against those measured with a cuff sphygmomanometer (N = 42). From left to right, the light source is blue (B), green (G), red (R), and near-infrared (NIR). The solid line and the dashed lines on each plot represent fixed bias (M) and M ± 1.96 standard deviation range, respectively.

**Figure 10:**
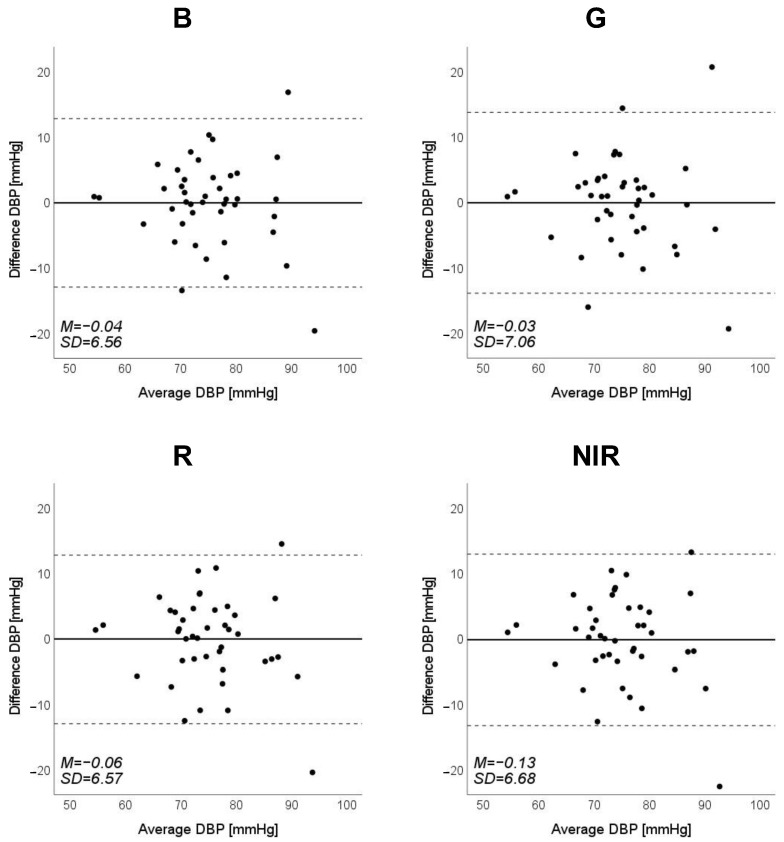
Bland–Altman plots of estimated diastolic blood pressure (DBP) against those measured with a cuff sphygmomanometer (*N* = 42). From left to right, the light source is blue (B), green (G), red (R), and near-infrared (NIR). The solid line and the dashed lines on each plot represent fixed bias (M) and M ± 1.96 standard deviation range, respectively.

**Figure 11:**
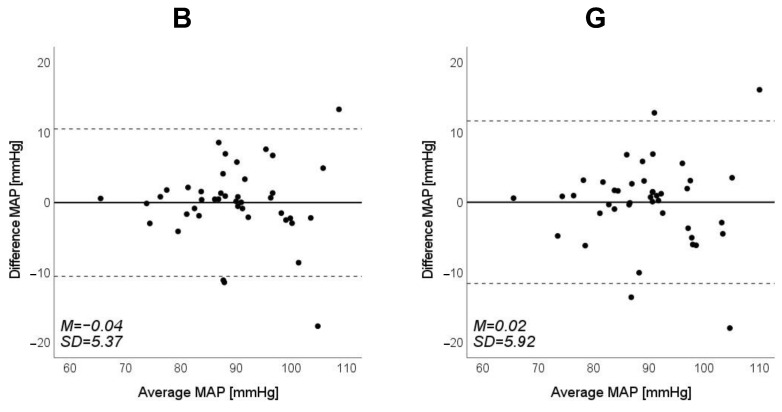

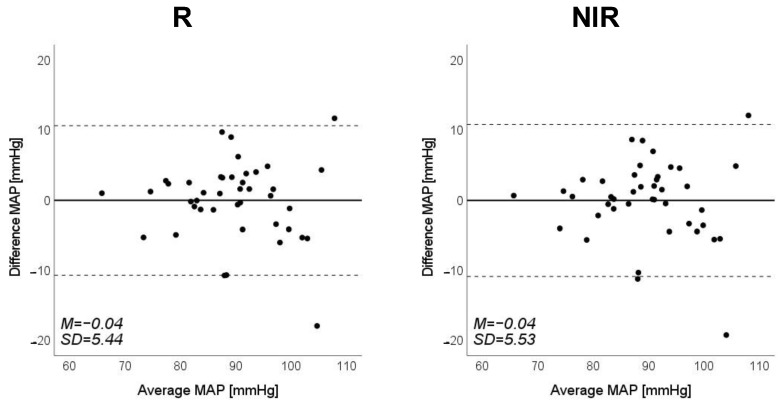
Bland–Altman plots of estimated mean arterial pressure (MAP) against those measured with a cuff sphygmomanometer (*N* = 42). From left to right, the light source is blue (B), green (G), red (R), and near-infrared (NIR). The solid line and the dashed lines on each plot represent fixed bias (M) and M ± 1.96 standard deviation range, respectively.

Scatter plots of the measured Δln SBP, measured Δln DBP, and measured Δln MAP obtained using the cuff sphygmomanometer and the estimated Δln SBP, estimated Δln DBP, and estimated Δln MAP at each wavelength are shown in Figure 6, Figure 7 and Figure 8, and Bland–Altman plots are shown in Figure 9, Figure 10 and Figure 11. In addition, Table 4 summarizes the means and standard deviations of the differences between the BP values measured with a cuff sphygmomanometer and the estimated BP values, and the number of data when the error is divided into 4 classes: 0–5, 5–10, 10–15, and >15 mmHg. Figure 9, Figure 10 and Figure 11 and Table 4 show that most of the values are within the limits of agreement, which indicates that the BP estimated using this method agrees well with the measured BP regardless of the wavelength.

Table 5 shows the means and standard deviations of each estimated BP from the PPG for each condition. Regardless of the wavelength, DBP, SBP, and MAP increased in the MA condition compared with BL. This difference in BP was statistically significant and was consistent with the trend of BP measured with a cuff sphygmomanometer. Therefore, changes in BP were captured using the BP estimated with this method.

## 4. Discussion

In Table 6, we focus on the wavelengths of the LEDs, having obtained the means of the *R*^2^ values for the three types of BP (DBP, SBP, and MAP) and compared them using a one-way repeated measures analysis of variance (ANOVA). For post hoc comparison, we used Tukey’s honestly significant difference (HSD) test for multiple comparisons. The one-way repeated measures ANOVA revealed the main effect of type, *F*(3,6) = 18.74, *p* = 0.002. The subsequent post hoc Tukey’s HSD test for multiple comparisons revealed that the mean *R*^2^ of green was less than that of blue, red, and NIR. This result indicates that wavelengths other than green should be used as the light source with this method. The inferiority of green LEDs is due to the low *R*^2^ of DBP, which is clearly dependent on the TPR [39]. Therefore, the measurement of the mNPV, which is correlated with the TPR, may lack accuracy. This may be due to a problem with the vessels that are captured at the depth of arrival of the green light.

Focusing on the *R*^2^ values in Table 3, we consider the optimal wavelengths for SBP, DBP, and MAP. For SBP, NIR is the optimal wavelength because of its largest *R*^2^ (=0.634) in the order of NIR > blue > red > green. Similarly, for DBP and MAP, the order is blue > NIR > red > green, so blue is the optimal wavelength (*R*^2^ = 0.497 for DBP and *R*^2^ = 0.630 for MAP). However, these differences were not statistically significant. Furthermore, we focus on the values of the standard partial regression coefficient (std. *β*) of the *β* coefficients for HR (*a*) and mNPV (*b*) at the optimal wavelengths (SBP: NIR; DBP and MAP: blue) in Table 3. For SBP, the coefficient *a* was larger than *b* for std. *β*. For DBP, *b* was larger than *a*. For MAP, *a* and *b* were about the same. Previous studies have shown that HR depends on CO in SBP [40,41], TPR in DBP [41], and CO and TPR in MAP [14]. Because CO and TPR are associated with HR and mNPV, respectively [28,29,30,31,32], the trend of std. *β*, whereby the *a* of HR is dominant in SBP and the *b* of mNPV is dominant in DBP, agrees well with physiological trends.

In this study, the optimal wavelengths for SBP, DBP, and MAP were different, which is likely because of the differences in the optical properties of biological tissues. The depth to which light penetrates biological tissue differs depending on wavelength, with longer-wavelength light penetrating deeper. Regarding the wavelengths used in this study, blue, green, red, and NIR reach deeper in that order, such that NIR can reach deep blood vessels, whereas blue can only reach peripheral ones in the epidermis. Therefore, using NIR gives information about relatively large blood vessels in deep areas, which have large changes in blood vessel volume, and the HR can be obtained with high accuracy; however, the information about peripheral blood vessels in the epidermis is lost among that about deep vessels. On the other hand, because blue reaches only the epidermis, it is thought that using it produces PPGs that reflect well the information about peripheral vessels. Therefore, we reason that NIR, which is better at measuring HR, and blue, which is better at measuring mNPV, can estimate SBP and DBP more accurately, respectively. For MAP, both blue and NIR are highly accurate, but in this study, at least among the participants before the experiment, the intensity of light emitted from the body was set to be constant at each wavelength, so the difference in the accuracy of the HR obtained from the PPG at each wavelength was lower while that of the blue estimation was higher. In other words, if a light source is available that can emit sufficiently strong light, then blue is the optimal wavelength for this method. Based on the above results, although no statistically significant difference was found for the trend of the present results, we believe that the results have a certain significance when physiological and optical characteristics are considered.

This study has several limitations. First, the participants were relatively few in number and included only healthy Japanese men and women of approximately the same age; therefore, the effects of the age, skin color, and health status of the participants require further investigation. Regarding the small number of data, we note that this study is at the stage of basic investigation and not at the stage of attempting practical application. Second, the participants were measured only while physically still, but it is known that PPGs are strongly affected by motion artifacts and that the effect varies depending on the wavelength of the light source [42]; therefore, for this method to be used in practice, it is necessary to investigate the extent to which the measurement accuracy is degraded and whether the optimum wavelength is changed in an environment in which body motion occurs. To address the small number of participants and the age bias in this study, in future work, it will be necessary to conduct experiments with a large number of participants and a wider range of ages to determine whether the results are statistically significant. Figure 6, Figure 7 and Figure 8 show that the error tends to be larger when blood pressure is higher. To realize the practical application of the proposed method, further improvements in accuracy are needed, including this point. If these problems can be solved, the proposed method is expected to be a useful method for performing simple blood pressure estimation.

Despite the above limitations, we have proposed a BP estimation method that requires only a simple PPG sensor, unlike a cuff sphygmomanometer or methods that require PPGs and ECGs. Furthermore, we have clarified the differences in the measurement accuracy due to the differences in light source wavelengths, which have not been considered to date.

## 5. Conclusions

In this study, we investigated the optimal light source wavelength for cuffless BP estimation using only the HR and mNPV obtained from PPGs. Using a PPG sensor capable of measuring PPGs simultaneously at four different wavelengths, we evaluated the accuracy of the estimation by performing multiple regression analysis on the HR and mNPV at each wavelength obtained for the SBP, DBP, and MAP measured both from the PPGs and using a cuff sphygmomanometer. From the results, although not significant, NIR was found to be the most accurate wavelength for SBP, and blue was found to be that for DBP and MAP. These results are consistent with physiological findings and indicate that the light source wavelength will become an important factor in cuffless BP estimation using PPGs in the future and that the accuracy of BP estimation can be improved by selecting the optimal wavelength according to the type of blood pressure.

## Figures and Tables

**Table 1:** Participants’ BPs measured with a cuff sphygmomanometer in each measurement condition.

Condition	SBP [mmHg]		DBP [mmHg]		MAP [mmHg]	
	Mean	SD	Mean	SD	Mean	SD
BL	112.3	12.3	68.5	6.3	83.1	7.2
MA	121.6	14.2	77.9	8.7	92.5	9.4

BP = blood pressure, SBP = systolic BP, DBP = diastolic BP, MAP = mean arterial pressure, mean = average of BP, SD = standard deviation of BP, BL = baseline (resting condition), MA = mental arithmetic (stress condition induced via mental arithmetic), and *N* = 14.

**Table 2:** Statistical results for HR and mNPV at 4 wavelengths in each condition.

Property	LED Color	Condition				Paired *t*-Test between BL and MA	
		BL		MA			
		Mean	SD	Mean	SD	*t*	*p*
HR [bpm]	Blue	72.8	12.7	89.8	15.8	−7.55	<0.001
	Green	72.8	12.7	89.5	15.7	−7.31	<0.001
	Red	72.7	12.5	88.6	15.2	−7.40	<0.001
	NIR	72.9	12.8	88.0	15.2	−6.45	<0.001
mNPV [a.u.]	Blue	0.0378	0.0179	0.0203	0.0074	6.28	<0.001
	Green	0.0364	0.0208	0.0193	0.0086	5.62	<0.001
	Red	0.0206	0.0104	0.0111	0.0041	4.80	<0.001
	NIR	0.0247	0.0115	0.0126	0.0047	5.84	<0.001

HR = heart rate, mNPV = modified normalized pulse volume, BL = baseline (resting condition), MA = mental arithmetic (stress condition induced via mental arithmetic), and *N* = 14.

**Table 3:** Results of multiple linear regression analysis of BP estimation at each wavelength.

BP	LED Color			HR (*a*)			mNPV (*b*)			*c*	
		*R* ^2^	*p*	*Β*	std. *β*	*p*	*β*	std. *β*	*p*	*β*	*p*
Δln SBP	Blue	0.612	<0.001	0.288	0.572	<0.001	−0.045	−0.262	0.079	−0.003	0.771
	Green	0.549	<0.001	0.337	0.661	<0.001	−0.022	−0.129	0.329	−0.003	0.816
	Red	0.592	<0.001	0.330	0.620	<0.001	−0.030	−0.209	0.122	−0.001	0.923
	NIR	0.634	<0.001	0.373	0.736	<0.001	−0.012	−0.086	0.520	0.001	0.914
Δln DBP	Blue	0.497	<0.001	0.203	0.280	0.097	−0.117	−0.475	0.006	0.024	0.168
	Green	0.406	<0.001	0.405	0.552	<0.001	−0.032	−0.134	0.375	0.021	0.267
	Red	0.48	<0.001	0.273	0.355	0.022	−0.084	−0.411	0.009	0.031	0.090
	NIR	0.47	<0.001	0.205	0.281	0.085	−0.097	−0.463	0.006	0.036	0.035
Δln MAP	Blue	0.630	<0.001	0.245	0.424	0.005	−0.084	−0.431	0.004	0.011	0.329
	Green	0.541	<0.001	0.377	0.646	<0.001	−0.028	−0.143	0.282	0.010	0.442
	Red	0.607	<0.001	0.303	0.497	<0.001	−0.059	−0.362	0.008	0.016	0.197
	NIR	0.598	<0.001	0.286	0.493	0.001	−0.058	−0.349	0.016	0.020	0.089

Δln BP = *a* × Δln (heart rate (HR)) + *b* × Δln (modified normalized pulse volume (mNPV)) + constant (*c*). BP = blood pressure, SBP = systolic BP, DBP = diastolic BP, MAP = mean arterial pressure, NIR = near-infrared, *R*^2^ = coefficient of determination, *β* = *β* coefficient of multiple linear regression, and std. *β* = standardized *β*.

**Table 4:** Difference between estimated BP and measured BP with a cuff sphygmomanometer.

	SBP				DBP				MAP			
	B	G	R	NIR	B	G	R	NIR	B	G	R	NIR
Mean (mmHg)	4.84	5.27	4.90	4.67	4.68	5.13	5.00	5.01	3.57	4.09	3.98	3.97
SD (mmHg)	3.83	4.20	4.06	3.79	4.60	4.84	4.25	4.42	4.01	4.28	3.71	3.86
0–5 mmHg	24	22	25	26	27	26	27	27	32	29	31	32
5–10 mmHg	12	15	13	13	10	11	8	10	6	8	7	6
10–15 mmHg	6	3	2	2	3	2	6	4	3	3	3	3
>15 mmHg	0	2	2	1	2	3	1	1	1	2	1	1

BP = blood pressure; SBP = systolic BP; DBP = diastolic BP; MAP = mean arterial pressure; B = blue LED; G = green LED; R = red LED; NIR = near-infrared LED; mean = average difference between measured BP and estimated BP; SD = standard deviation of difference between measured BP and estimated BP; and 0–5, 5–10, 10–15, and >15 mmHg: number of data when the error is divided into 4 classes.

**Table 5:** Statistical results of estimated BP at 4 wavelengths in each condition.

Property	LED Color	Condition				Paired *t*-Test between BL and MA	
		BL		MA			
		Mean	SD	Mean	SD	*t*	*p*
SBP [mmHg]	Blue	111.6	13.2	122.3	14.4	−7.85	<0.001
	Green	112.0	13.4	122.1	14.3	−8.16	<0.001
	Red	111.9	13.2	122.2	13.9	−7.69	<0.001
	NIR	112.2	13.2	122.0	14.0	−6.85	<0.001
DBP [mmHg]	Blue	69.3	6.5	78.0	7.8	−7.22	<0.001
	Green	69.8	6.6	77.8	8.2	−7.89	<0.001
	Red	69.4	6.1	77.9	7.5	−7.38	<0.001
	NIR	69.3	6.2	77.9	7.3	−8.21	<0.001
MAP [mmHg]	Blue	83.4	7.6	92.7	8.9	−7.62	<0.001
	Green	83.9	7.7	92.6	9.0	−8.07	<0.001
	Red	83.5	7.3	92.7	8.3	−7.67	<0.001
	NIR	83.6	7.6	92.6	8.5	−8.16	<0.001

BP = blood pressure, SBP = systolic BP, DBP = diastolic BP, MAP = mean arterial pressure, BL = baseline (resting condition), MA = mental arithmetic (stress condition induced via mental arithmetic), and *N* = 14.

**Table 6:** Coefficient of determination at each wavelength of PPG and results of statistical tests.

	LED Color								Results of Statistical Tests		
	B		G		R		NIR		ANOVA		Tukey’s HSD Multiple Comparison
	Mean	SD	Mean	SD	Mean	SD	Mean	SD	*F*(3,6)	*p*	
*R* ^2^	0.580	0.059	0.499	0.066	0.560	0.057	0.567	0.070	18.74	0.002	G < B, R, NIR

*R*^2^ = coefficient of determination, B = blue LED, G = green LED, R = red LED, NIR = near-infrared LED, and HSD = honestly significant difference.

## Data Availability

The data supporting the findings of this study, except for the raw data, are available via e-mail from the corresponding author upon reasonable request.
